# Human pancreatic cancer xenografts recapitulate key aspects of cancer cachexia

**DOI:** 10.18632/oncotarget.13593

**Published:** 2016-11-25

**Authors:** Daniel Delitto, Sarah M. Judge, Andrea E. Delitto, Rachel L. Nosacka, Fernanda G. Rocha, Bayli B. DiVita, Michael H. Gerber, Thomas J. George, Kevin E. Behrns, Steven J. Hughes, Shannon M. Wallet, Andrew R. Judge, Jose G. Trevino

**Affiliations:** ^1^ Department of Surgery, College of Medicine, University of Florida Health Science Center, Gainesville, FL 32610, USA; ^2^ Department of Physical Therapy, University of Florida Health Science Center, Gainesville, FL 32610, USA; ^3^ Department of Oral Biology, College of Dentistry, University of Florida Health Science Center, Gainesville, FL 32610, USA; ^4^ Department of Medicine, College of Medicine, University of Florida Health Science Center, Gainesville, FL 32610, USA

**Keywords:** pancreatic cancer, cachexia, muscle wasting, inflammation, xenografts

## Abstract

Cancer cachexia represents a debilitating syndrome that diminishes quality of life and augments the toxicities of conventional treatments. Cancer cachexia is particularly debilitating in patients with pancreatic cancer (PC). Mechanisms responsible for cancer cachexia are under investigation and are largely derived from observations in syngeneic murine models of cancer which are limited in PC. We evaluate the effect of human PC cells on both muscle wasting and the systemic inflammatory milieu potentially contributing to PC-associated cachexia. Specifically, human PC xenografts were generated by implantation of pancreatic cancer cells, L3.6pl and PANC-1, either in the flank or orthotopically within the pancreas. Mice bearing orthotopic xenografts demonstrated significant muscle wasting and atrophy-associated gene expression changes compared to controls. Further, despite the absence of adaptive immunity, splenic tissue from orthotopically engrafted mice demonstrated elevations in several pro-inflammatory cytokines associated with cancer cachexia, including TNFα, IL1β, IL6 and KC (murine IL8 homologue), when compared to controls. Therefore, data presented here support further investigation into the complexity of cancer cachexia in PC to identify potential targets for this debilitating syndrome.

## INTRODUCTION

With mortality rates largely unchanged over 50 years, pancreatic cancer (PC) is projected to be the second leading cause of cancer deaths by 2030 [1, 2]. Most patients who present with PC do so with a recent history of weight loss, anorexia, malaise and an acute phase response, better known as cancer cachexia [3].

Patient debilitation is often so severe that the off-target effects of systemic chemotherapies or radiation can have additive and devastating consequences of rapid deconditioning, further muscle catabolism and death prior to any therapeutic benefit [4, 5]. Thus, many patients with PC ultimately succumb from complications associated with cancer cachexia [6]. Therefore, identifying potential targets to interrupt the signaling cascade contributing to cancer cachexia may reduce debilitation/morbidity and, as a result, allow for the implementation of more effective treatments in PC.

Cancer cachexia is a complex metabolic syndrome characterized by the loss of muscle mass that cannot be reversed by nutritional support alone [7–11]. The muscle wasting observed in cachexia is associated with a wide array of molecular pathways, some of which are triggered by tumor and host-derived systemic inflammatory changes in advanced cancer [12]. Many of these pathways converge on the inappropriate activation of the ubiquitin-proteasome system (UPS), leading to increased degradation of muscle proteins and progressive loss of contractile machinery [13, 14]. Investigation therefore focuses on systemic signals that drive UPS activation in myocytes, which has yielded numerous potential therapeutic targets [15]. However, clinical trials have demonstrated that many of these targets in isolation, such as secreted cytokines, are not solely responsible for sustaining cancer-induced cachexia [4, 16]. To complicate matters, it is unclear why severe cachexia is observed in some patients while others are relatively spared.

PC represents a heterogeneous disease with respect to cachexia, and tumor burden alone does not predict the severity of muscle wasting [17, 18]. Factor(s) responsible for this heterogeneity likely stem from differences in the tumor and host response. Therefore, separating tumor-specific and host-specific factors that contribute to cachexia may delineate therapeutic targets and patient populations likely to benefit from these therapies. However, these targets remain difficult to elucidate unless experimental models evolve to better recapitulate the human disease. To address this need, we evaluated skeletal muscle wasting along with key components of the host inflammatory response in human PC xenograft models, building upon findings from syngeneic murine PC models [19, 20] and preliminary observations in human xenograft models [21, 22]. The growth of human PC cells induced significant muscle wasting along with gene expression profiles consistent with muscle catabolism. We further define a systemic inflammatory profile associated with this model of PC cachexia. Taken together our data suggest that human PC xenografts represent a valuable experimental tool to evaluate PC-associated cachexia and associated therapeutics.

## RESULTS

### L3.6pl subcutaneous and orthotopic xenografts induce cancer cachexia

**Figure 1:**
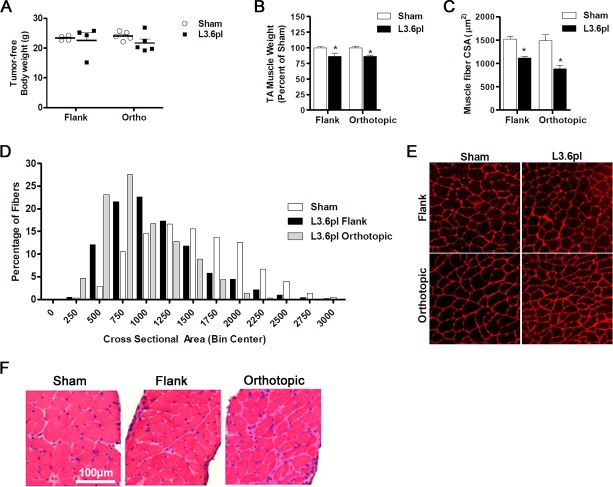
Flank and orthotopic L3.6pl xenografts induce cancer cachexia **A.** Tumor-free body weight and muscle mass **B.** of tibialis anterior (TA) muscles harvested from NSG mice bearing L3.6pl xenografts in the flank and orthotopically in the pancreas compared to Sham. **C-E.** Cross-sections of TA muscles were stained with wheat germ agglutinin (red) to visualize muscle fiber membranes and the average fiber cross sectional area (CSA) was calculated for each group (C). (D) Fiber CSA data are further presented as a frequency distribution to demonstrate the relative distribution of fiber sizes for each group. (E) Representative images of muscle cross-sections. **F.** Representative H&E sections of diaphragm muscle are displayed for mice bearing flank and orthotopic L3.6pl xenografts compared to sham. Data represent mean ± SE. *P < 0.05 vs sham control group using the Mann-Whitney U test.

We first determined if human PC xenografts placed subcutaneously in the flank or orthotopically in the pancreas were able to induce body weight loss and skeletal muscle wasting. Tissues from mice bearing L3.6pl PC cell flank tumors and respective sham control mice were harvested approximately 4 weeks following tumor cell inoculation. Similarly, mice bearing L3.6pl orthotopic tumors and sham controls were sacrificed and tissues harvested between 4-6 weeks following tumor cell inoculation. Timing of tissue harvest was based upon tumor endpoint criteria, consisting of both tumor size and body condition. Tumor weights were not significantly different between flank and orthotopic tumors at endpoint (2.7±0.3 g vs. 3.3±0.2 g; *P* = 0.14). No significant differences were found between the body weight of sham mice and the tumor-free body weight of mice bearing L3.6pl flank or orthotopic tumors (Figure 1A). Despite this, significant skeletal muscle wasting was evident in both L3.6pl flank and orthotopic tumor-bearing groups when compared to sham controls, as determined by tibialis anterior (TA) muscle weight and fiber cross sectional area (CSA) (Figure 1C–1D). TA muscles from mice bearing L3.6pl flank tumors weighed 14% less than sham, while TA muscles from mice bearing L3.6pl orthotopic tumors weighed 15% less than sham. Further, a 27% decrease in fiber CSA was observed in mice bearing L3.6pl flank tumors, and a 40% decrease in muscle fiber CSA was observed in mice bearing orthotopic tumors (Figure 1D–1E). We also observed atrophy in the triceps surae muscle group (gastrocnemius, soleus and plantaris muscles), which weighed 13% less in mice bearing L3.6pl flank tumors compared to sham controls (111±6.7 mg vs. 128±2.6 mg; *P* = 0.09) and 19% less in mice bearing L3.6pl orthotopic tumors compared to sham controls (102±4.3 mg vs. 126±2.6 mg; *P* < 0.01). We also performed qualitative H&E analyses on TA (data not shown) and diaphragm muscles from all groups. As expected, muscle fibers were visually smaller in both the TA and diaphragm of tumor-bearing groups compared to sham. However, we also noted additional muscle pathologies specifically in the diaphragm muscle of mice bearing orthotopic L3.6pl xenografts, but not mice bearing flank xenografts. Indeed, diaphragms from all orthotopic L3.6pl tumor-bearing mice showed increased extracellular space surrounding muscle fibers, greater variation in fiber shape, and increased presence of mononuclear cells compared to sham mice (Figure 1F). Thus, L3.6pl xenografts induce significant muscle wasting regardless of tumor microenvironment, though our data suggest that tumors located orthotopically in the pancreas may result in more significant muscle pathology.

### Orthotopically implanted PANC-1 cells induce cancer cachexia

**Figure 2:**
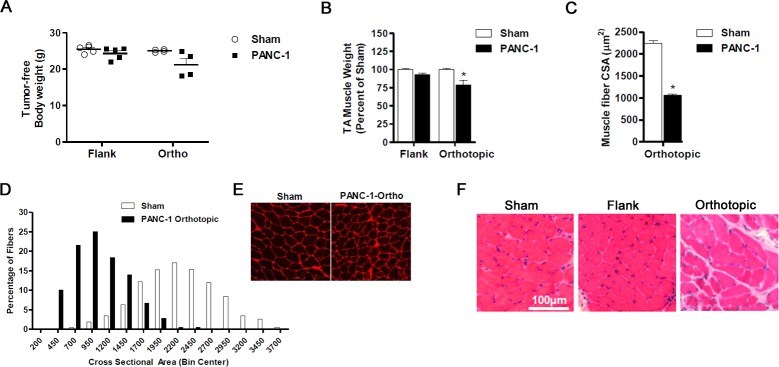
Orthotopic PANC-1 xenografts induce cancer cachexia **A.** Tumor-free body weight and muscle mass **B.** of tibialis anterior (TA) muscles harvested from NSG mice bearing PANC-1 xenografts in the flank and orthotopically in the pancreas compared to Sham. **C-E.** Cross-sections of TA muscles from Sham and PANC-1 orthotopic groups were stained with wheat germ agglutinin (red) to visualize muscle fiber membranes and the average fiber cross sectional area (CSA) was calculated for each group (C). (D) Fiber CSA data are further presented as a frequency distribution to demonstrate the relative distribution of fiber sizes for each group. (E) Representative images of muscle cross-sections. **F.** Representative H&E sections of diaphragm muscle are displayed for mice bearing flank and orthotopic PANC-1 xenografts compared to sham. Data represent mean ± SE. *P < 0.05 vs sham control group using the Mann-Whitney U test.

As previously mentioned, the heterogeneity of PC cachexia may stem from differences in the tumor itself. We therefore evaluated the human PC xenograft model incorporating a different PC cell line, PANC-1. Tumor endpoint was reached for both flank and orthotopic groups at 10 weeks following tumor cell inoculation. Tumor weights were not significantly different between flank and orthotopic xenografts (1.39±0.1 g vs. 1.78±0.6 g; *P* = 0.90). No significant difference was found between the body weight of sham mice and the tumor-free body weight of mice bearing PANC-1 flank tumors (Figure 2A) nor were there significant differences in TA muscle weight (Figure 2B). However, the triceps surae muscle complex weighed 12% less than sham controls (124±4.2 mg vs. 141±2.5 mg; *P* < 0.01). In contrast, significant cachexia was observed in mice bearing PANC-1 orthotopic tumors. The tumor-free body weight of mice bearing PANC-1 orthotopic tumors was approximately 15% less than sham, while the TA muscle weighed 21% less (Figure 2C) and the triceps surae muscle complex weighted 17% less than sham (115±12.4 mg vs. 138±2.0 mg; *P* = 0.34). This loss of TA muscle mass in mice bearing PANC-1 orthotopic tumors translated to a ~50% decrease in the average TA muscle fiber CSA compared to sham (Figure 2D–2E). Since the mass of TA muscles from mice bearing PANC-1 flank tumors was not statistically different from sham mice, the average muscle CSA was not measured in these groups. Diaphragm histology revealed findings comparable to those observed in the L3.6pl model. Indeed, while fiber atrophy was evident in mice bearing PANC-1 flank or orthotopic xenografts, orthotopic xenografts resulted in a more severe muscle pathology (Figure 2F). Taken together, mice bearing orthotopic PANC-1 xenografts demonstrated more findings consistent with cancer cachexia compared to those with flank xenografts, suggesting the tumor microenvironment may contribute to the development and/or rate of tumor-induced muscle wasting.

### L3.6pl xenografts induce changes in muscle atrophy-related transcription factors

**Figure 3:**
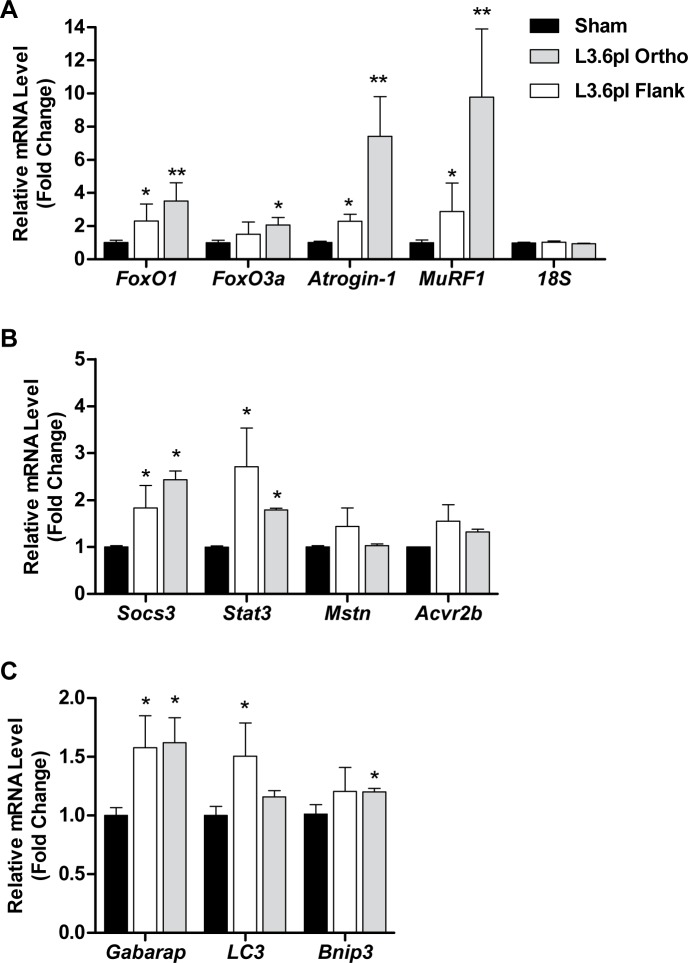
L3.6pl xenografts induce changes in muscle atrophy-related biomarkers. Gene expression of FoxO1, FoxO3a, atrogin-1 and MuRF1 **A.**, Stat3, Socs3, myostatin (Mstn) and activin receptor 2b (Acvr2b) **B.** or Gabarap, Lc3 and Bnip3 **C.** was quantified from tibialis anterior (TA) muscles harvested from L3.6pl tumor-bearing mice using qRT-PCR and normalized to 18S. *P < 0.05 vs sham control group using the Mann-Whitney U test. **P < 0.01 vs sham control group using the Mann-Whitney U test.

We further determined whether the muscle atrophy evident in L3.6pl tumor-bearing mice was associated with increased gene expression of muscle atrophy-related transcription factors and atrophy-related biomarkers previously implicated in cancer-related muscle wasting. Specifically, we measured the gene expression of the Forkhead Box O (FoxO) transcription factors FoxO1 and FoxO3, the muscle-specific ubiquitin E3 ligases, atrogin-1/MAFbx/Fbxo32 and MuRF1/Trim63, Stat3 and its target gene Socs3, Myostatin (Mstn) and its receptor, activin receptor 2b (Acvr2b) and autophagy-related genes Gabarap, Lc3 and Bnip3. As shown in Figure 3, the expression of FoxO1, atrogin-1, MuRF1, Stat3, Socs3 and Gabarap was significantly elevated in the muscle of both L3.6pl flank and orthotopic tumor-bearing groups when compared to sham, while FoxO3, Bnip3 and Acvr2b expression was significantly elevated in only the L3.6pl orthotopic tumor-bearing group. Lc3 was significantly elevated in only L3.6pl flank tumor-bearing group. Neither flank nor orthotopic L3.6pl tumor-bearing groups presented with a statistically significant increase in Mstn. Due to the time difference in which tumor endpoint was reached between mice bearing L3.6pl flank and orthotopic tumors, we are unable to make direct statistical comparisons between the orthotopic and flank tumor-bearing groups. However, changes in the expression of atrophy-related genes were clearly more robust in response to the orthotopic tumors, which is in agreement with the fiber CSA data.

### L3.6pl xenografts induce pro-inflammatory cytokines implicated in cachexia

**Figure 4:**
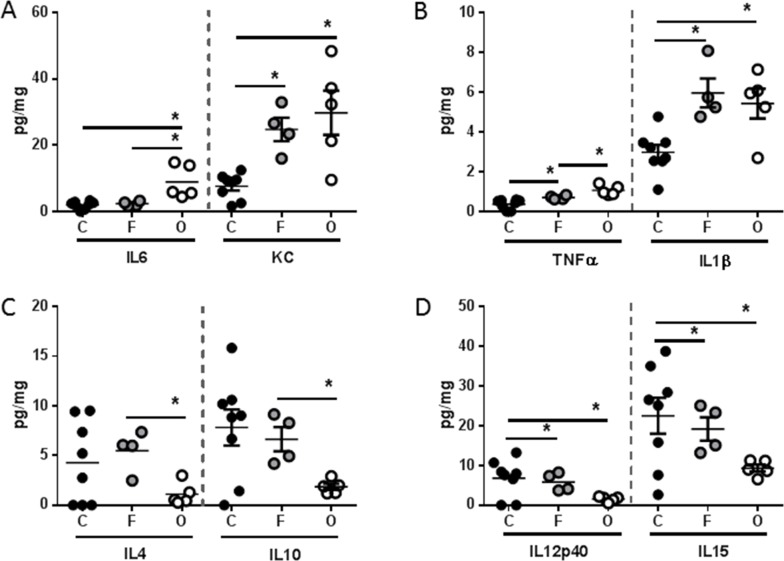
L3.6pl tumors placed subcutaneously or orthotopically induce a pro-inflammatory systemic environment Spleens were isolated from L3.6pl flank (F) or orthotopic (O) tumor-bearing mice as well as sham controls (C), and homogenized. After which, **A.** IL6, KC, **B.** TNFα, IL1β, **C.** IL4, IL10, and **D.** IL12p40, IL15 were probed for using multiplex technology. *P value <0.05 using one-way ANOVA followed by Mann Whitney U test for multiple comparisons.

Pro-inflammatory cytokines have long been implicated as a driving force behind cachexia [12]. Thus, we evaluated the splenic expression of a panel of soluble mediators. Several pro-inflammatory cytokines were found at higher concentrations in the spleen of both flank and orthotopic tumor-bearing groups when compared to sham, including KC (murine IL8 homologue), TNFα, and IL1β (Figure 4A–4B). Interestingly, IL6 was found at higher concentrations only in the orthotopic tumor-bearing group (Figure 4A). Conversely, the anti-inflammatory cytokines IL4 and IL10 were found at significantly lower concentration in the spleen of only the orthotopic tumor-bearing group when compared to sham (Figure 4C). Finally, cytokines associated with T helper cell (Th) polarization, IL12p40 and IL15 were also found at lower concentrations in the spleen of both flank and orthotopic tumor-bearing groups when compared to sham (Figure 4D).

**Figure 5:**
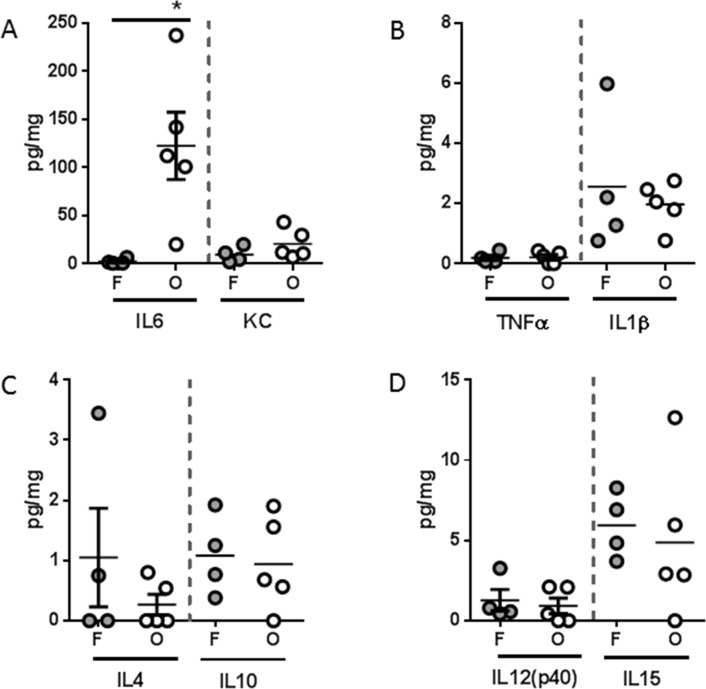
Tumor inflammatory microenvironment of L3.6pl tumors placed subcutaneously or orthotopically Tumors were isolated from L3.6pl flank (F) or orthotopic (O) tumor-bearing mice and homogenized. After which, **A.** IL6, KC, **B.** TNFα, IL1β, **C.** IL4, IL10, and **D.** IL12p40, IL15 were probed using multiplex technology. *P value <0.05 using one-way ANOVA followed by Mann Whitney U test for multiple comparisons.

As it has been suggested that the tumor microenvironment can shape the immune micro-environment, the same immune mediators were also evaluated in tumor lysates. All mediators detected systemically were either undetectable or detected at very low levels within the tumor lysates. In addition, there was no statistical difference in the concentrations of any mediators, except IL6, comparing lysates from tumors grown in the flank to those grown orthotopically (Figure 5). Together these data suggest that growth of L3.6pl tumors subcutaneously in the flank or orthotopically in the pancreas results in induction of a pro-inflammatory systemic environment, whereby the changes are most robust in orthotopic tumor-bearing mice. Importantly, these systemic responses are most likely not due simply due to a robust pro-inflammatory tumor immune microenvironment.

### Orthotopic PANC-1 xenografts demonstrate changes in muscle atrophy-related transcription factors and induce cachexia-associated pro-inflammatory cytokines

**Figure 6:**
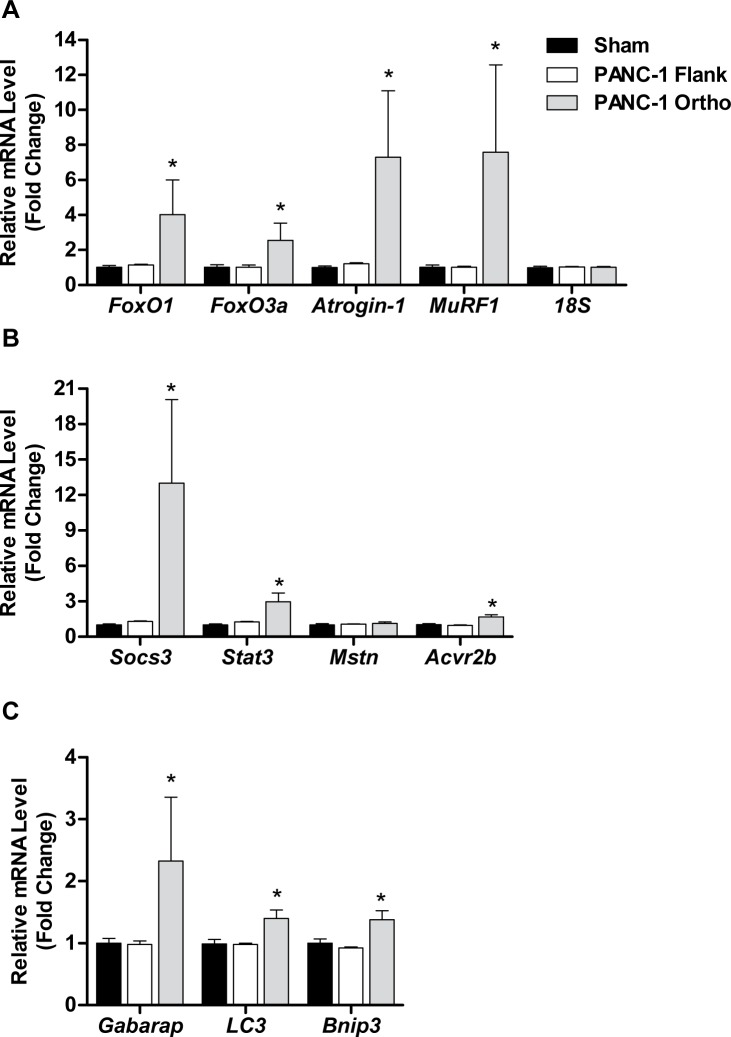
Orthotopic PANC-1 xenografts induce changes in muscle atrophy-related transcription factors Gene expression of FoxO1, FoxO3a, atrogin-1 and MuRF1 **A.**, Stat3, Socs3, myostatin (Mstn) and activin receptor 2b (Acvr2b) **B.** or Gabarap, Lc3 and Bnip3 **C.** was quantified from tibialis anterior (TA) muscles harvested from L3.6pl tumor-bearing mice using qRT-PCR and normalized to 18S. *P < 0.05 vs sham control group using the Mann-Whitney U test. **P < 0.01 vs sham control group using the Mann-Whitney U test.

Gene expression analyses of TA muscles from PANC-1 flank and orthotopic tumor-bearing groups and their respective sham groups revealed findings consistent with the muscle weight data. Specifically, mice bearing PANC-1 flank tumors showed no significant increases in any of the atrophy-related genes measured, while mice bearing PANC-1 orthotopic tumors showed robust increases in nearly all of the atrophy genes measured, including FoxO1, FoxO3, atrogin-1, MuRF1, Stat3, Socs3, Acvr2b, Gabarap, Lc3 and Bnip3 (Figure 6). Thus, orthotopic growth of PANC-1 tumor cells within the pancreas, but not subcutaneous growth on the flank, causes significant muscle wasting that appears to be mediated, at least in part, by canonical atrophy signaling pathways previously implicated in cancer-related muscle wasting.

**Figure 7:**
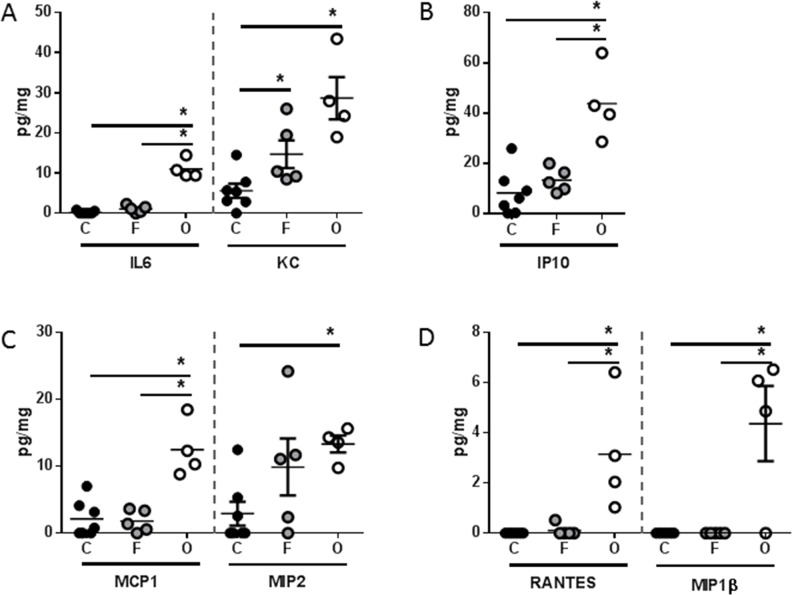
PANC-1 tumors placed subcutaneously or orthotopically induce a unique pro-inflammatory systemic environment Spleens were isolated from PANC-1 flank (F) or orthotopic (O) tumor-bearing mice as well as sham controls (C), and homogenized. After which, **A.** IL6, KC, **B.** IP10 **C.** MCP1, MIP2, (D) RANTES, and MIP1β were probed for using multiplex technology. *P value <0.05 using one-way ANOVA followed by Mann Whitney U test for multiple comparisons.

**Figure 8:**
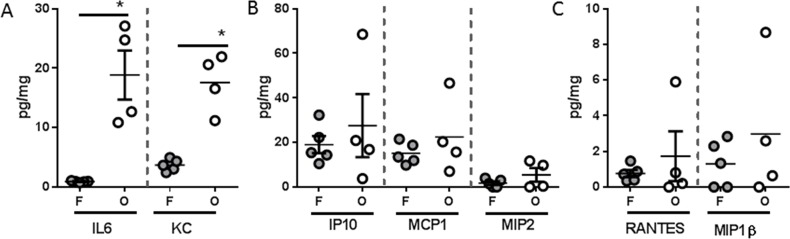
Tumor inflammatory microenvironment of PANC-1 tumors placed subcutaneously or orthotopically Tumors were isolated from PANC-1 flank (F) or orthotopic (O) tumor-bearing mice and homogenized. After which, **A.** IL6, KC, **B.** IP10 **C.** MCP1, MIP2, **D.** RANTES, and MIP1β were probed for using multiplex technology. *P value <0.05 using one-way ANOVA followed by Mann Whitney U test for multiple comparisons.

Analysis of systemic immune mediators also revealed significant differences between PANC-1 flank and orthotopic tumor-bearing groups, albeit with a distinctly different soluble mediator profile than was observed with L3.6pl tumor-bearing mice (Figure 7). Specifically, higher concentrations of only the pro-inflammatory cytokine KC (murine IL8 homologue) was found in the spleen of both PANC-1 flank and orthotopic tumor-bearing groups when compared to sham, but only orthotopic tumor-bearing mice presented with higher concentrations of IL6 (Figure 7A). In addition, the chemokines, IP10, MCP1, MIP2, RANTES and MIP1β were also found in higher concentrations in the spleen of orthotopic tumor-bearing mice when compared to sham as well as the PANC-1 flank tumor-bearing group (Figure 7B–7D). As was observed with L3.6pl tumor bearing mice, all mediators detected systemically were detected at very low levels within the lysates of PANC-1 flank and orthotopic tumors. Again there was no statistical difference in the concentrations of most mediators when comparing lysates from tumors grown in the flank to those grown orthotopically. However, higher concentrations of both IL6 and KC (murine IL8 homologue) were observed in PANC-1 orthotopic tumors (Figure 8). Together these data suggest that orthotopic growth of PANC-1 tumor cells within the pancreas, but not subcutaneous growth in the flank, induced systemic inflammation distinct from that observed in L3.6pl tumor-bearing mice. Most importantly, these data mirror that of the body and TA weight, as well as the atrophy gene data, again implicating systemic inflammation in mechanisms of muscle wasting and cachexia.

## DISCUSSION

Cachexia is a debilitating consequence of pancreatic cancer that diminishes quality of life and precludes effective systemic therapy [23–25]. Insights into targetable mechanisms of cancer cachexia have been largely developed from immunocompetent mouse models incorporating syngeneic cancer cell lines. While many inroads have been made in murine models of other neoplasms, conclusions derived from investigations employing mouse models of colon, lung and skin cancer may not necessarily translate to PC [3, 24]. In addition, consistent use of a relatively small pool of murine PC tumor cell lines may suffer in its applicability to the human disease. Our data demonstrate that the use of two different human PC cell lines resulted in muscle wasting and systemic inflammatory profiles consistent with cancer cachexia. However, distinctions were observed between tumors derived from L3.6pl versus PANC-1. While the factors responsible for these differences are speculative at this point, these data reinforce clinical observations that the degree of muscle wasting is not solely dependent on tumor burden. Rather, the molecular properties of the tumor play a critical role. We also observed findings associated with advanced cachexia in tumor-bearing mice demonstrating little to no weight loss, supporting clinical observations that weight loss represents a late finding in cancer cachexia [4]. This work further reinforces the importance of orthotopic implantation in evaluating a systemic response. Regardless of cell line engrafted, orthotopic tumors induced a more robust cachectic phenotype, which should guide future studies investigating xenograft models of cancer cachexia [26].

Mechanisms implicated in cancer cachexia are diverse, indicative of its multifactorial nature [15, 27– 29]. Several molecular signaling pathways within the myocyte have been implicated in driving tumor-induced muscle wasting, including forkhead boxO (FoxO), signal transducer and activator of transcription 3 (STAT3), and myostatin-activin receptor 2B [30]. In the current study we used qRT-PCR to measure components of these pathways, as well as the atrophy biomarkers atrogin-1/Fbxo32 and MuRF1/Trim63, to determine whether the response of muscle to human PC cells is similar to syngeneic murine models of cancer cachexia. While limited by evaluation in a single muscle group, the response of muscle in the current study is nonetheless similar to previously published cachexia models [31], further demonstrating the potential importance of FoxO and its activation of genes involved in muscle protein breakdown, STAT3 and myostatin signaling in pancreatic cancer cachexia.

While murine lymphocyte and natural killer cell function must be sacrificed in order to engraft human cancer cells, the innate immune system of the NSG mouse remains otherwise intact [32, 33]. The immune status of the tumor-bearing host is particularly relevant because a systemic pro-inflammatory immune response contributes to cancer cachexia [15, 27]. Thus, the induction of cancer cachexia in our immunocompromised model suggests that the secretion of soluble mediators associated with innate immunity is sufficient to drive muscle wasting. Indeed, several pro-inflammatory cytokines were elevated systemically in both L3.6pl and PANC-1 flank and orthotopic xenograft models. In addition, like that of muscle wasting, the most robust phenotype was observed in the orthotopic xenograft model. Most strikingly, L3.6pl and PANC-1 each induced a unique profile of soluble mediators with L3.6pl tumor-bearing mice demonstrating a more cytokine-centric profile and PANC-1 tumor-bearing mice demonstrating a more chemokine-centric profile. These data again highlight that differences in PC cell biology contribute to unique immunological interactions. Indeed, while mice bearing L3.6pl or PANC-1 tumors were associated with distinct immunological phenotypes, both cohorts demonstrated muscle wasting, suggesting that multiple PC-induced systemic signals can result in convergent cachectic outcomes. In addition, all mediators detected systemically were not detectable within the tumor, suggesting that the systemic levels were not simply due to a pro-inflammatory tumor microenvironment or tumor extension into splenic tissue, which we did not observe in either model. Taken together, these data parallel phenomena observed in the human disease, as heterogeneity in the severity of PC cachexia cannot be explained by tumor burden alone [24, 34, 35].

In summary, we present an experimental model of cancer cachexia incorporating human cancer cells in NSG mice that consistently recapitulates key aspects of muscle wasting and systemic inflammation associated with the human disease. These findings support the use of this model in evaluating other aspects of cancer cachexia, such as anorexia, metabolic derangements and changes in adipose tissue. Additionally, validation of this experimental system with human cell lines supports further investigation with more applicable models, such as patient-derived xenografts, which serve to better recapitulate the pancreatic tumor microenvironment [36] and allow for personalized interventions targeting cancer cachexia [37]. Observations from this study will guide the incorporation of a validated patient-derived xenograft (PDX) model [26] into cancer cachexia, specifically highlighting the importance of orthotopic implantation. The PDX model also allows investigators to compare muscle wasting and systemic inflammation in patients to their PDX “avatars”, confirming these phenomena are not limited to the mouse model. Data presented here provide a foundation for PDX incorporation and therefore represent an important step toward improving experimental models of PC cachexia.

## MATERIALS AND METHODS

### Pancreatic cancer (PC) cell lines

The human pancreatic cancer cell line, PANC-1, was obtained from American Type Culture Collection ATCC (Rockville, MD). The humanL3.6pl metastatic variant was derived from repeated *in vivo* cycle of injecting primary pancreatic cancer cell line COLO-357 cells into the pancreas of nude mice, and selecting for liver metastases [38, 39]. All human PC cell lines were authenticated within 6 months by short tandem repeat (STR) analysis.

### Murine xenografts

All animal studies were performed with approval from the University of Florida Institutional Animal Care and Use Committee. Flank tumors were engrafted by subcutaneous injection of 10^6^ cancer cells embedded in 200 μL of 50% Matrigel® matrix (Corning, NY) into 8-week-old female NOD-SCID IL2 receptor gamma chain knockout (NSG) mice (Jackson Laboratory, Bar Harbor, ME). Alternatively, orthotopic injections were performed using 10^6^ pancreatic cancer cells embedded in 50 μL of 50% Matrigel® into the pancreas using a surgical technique described previously [40]. Control groups consisted of equal volumes of 50% Matrigel® matrix injected into age- and gender-matched NSG mice. Mice were anesthetized using inhaled isoflurane during the procedure and administered two doses of buprenorphine immediately and 12 hours postoperatively. Flank tumors were allowed to reach an endpoint of 2 cm in maximum diameter prior to euthanasia. Mice with orthotopic xenografts were euthanized when palpable intra-abdominal tumors reached 1 cm in size.

### Tissue harvest

At the time of euthanasia, bilateral tibialis anterior, gastrocnemius complexes and diaphragms were harvested for histologic and molecular analyses from tumor-bearing mice or age- and gender-matched control mice that underwent equivalent sham injections. Additionally, tumors and spleens were isolated and homogenized using bead beating in protein lysis buffer containing protease inhibitors.

### Muscle removal and processing

Diaphragms, triceps surae muscle complexes (gastrocnemius, soleus and plantaris) and tibialis anterior muscles were dissected from anesthetized mice, rinsed in PBS, weighed and snap frozen in liquid nitrogen for RNA isolation or embedded in OCT medium within a tissue embedding cassette prior to freezing in liquid nitrogen-cooled isopentane for cryosectioning. Using a Microm HM 550 Cryostat (Microm International, Walldorf, Germany), 10μM sections were cut from the midbelly of TA muscles and transferred to glass slides. For measurement of cross-sectional area, sections were fixed for 5 minutes in 4% paraformaldehyde, washed in 1xPBS and incubated with Alexa Fluor-conjugated wheat germ agglutinin (Invitrogen) for 1hr to visualize muscle fiber membranes. Images were captured using a Leica DM5000B microscope (Leica microsystems, Bannockburn, IL, USA) and the muscle fiber cross sectional area (CSA) of at least 250 muscle fibers per muscle was measured using Leica Application Suite 3.5.0 software. Hematoxylin and eosin (H&E) staining was also performed on fresh muscle cryosections from TA and diaphragm muscles immediately following cryosectioning as previously described [41].

### Gene expression profile

Tibialis anterior muscles were quickly minced and homogenized in Trizol using a polytron homogenizer, and RNA isolated as previously described [31]. RNA purity and concentration were determined through absorbance spectrophotometry at 230, 260 and 280 nm. cDNA was subsequently synthesized from equal amounts of RNA using Ambion's RETROscript FirstStrand Synthesis Kit (Life Technologies, Grand Island, NY). qRT-PCR was performed as previously described using a 7300 real-time PCR system and TaqMan^®^ Gene Expression Assays from Applied Biosytems for FoxO1 (NM_019739.3), FoxO3 (NM_019740.2), atrogin-1/MAFbx/Fbxo32 (NM_026346.2), MuRF1/Trim63 (NM_001039048.2), Stat3 (NM_011486.4), Socs3 (NM_007707.3), Mstn (NM_010834.2), Acvr2b (NM_007397.2), Gabarap (NM_019749.4), Lc3 (NM_025735.3), Bnip3 (NM_009760.4), and 18S (X03205.1).

### Soluble mediator analysis

Homogenates from tumors and spleens were probed for soluble mediators using the Milliplex® Premixed 25-Plex Mouse Immunology Multiplex Assay (Merck Millipore, Darmstadt, Germany) according to the manufacturer's protocol. Specifically, supernatants from tissue homogenates were incubated in filter bottom microtiter plates (EMD Millipore, San Jose, CA) with beads coated with primary antibodies overnight at 4C. After washing, PE-conjugated anti-cytokine antibodies were added and incubated for additional 2 hours at room temperature. Following washing, data was acquired on a Luminex 200 (EMD Millipore, San Jose, CA) and analyzed with Milliplex Software (EMD Millipore, San Jose, CA). Concentrations were quantified using a standard curve and 5 parameter logistics to determine pg/mL concentrations. All cytokine concentrations were normalized to total protein concentrations using detergent compatible protein quantification (Bio-Rad, Hercules, CA). Soluble mediator concentrations were then converted to pg/mg of tissue as follows: pg/ml divided by mg/ml of total protein.

### Statistics

Data were analyzed using a Mann Whitney U test, comparing each tumor-bearing group to their respective sham group (GraphPad Prism). Significance was established as *P* <0.05.
